# Retraction Note: Associations between VDR Gene Polymorphisms and Osteoporosis Risk and Bone Mineral Density in Postmenopausal Women: A systematic review and Meta-Analysis

**DOI:** 10.1038/s41598-021-88654-1

**Published:** 2021-04-21

**Authors:** Liang Zhang, Xin Yin, Jingcheng Wang, Daolinag Xu, Yongxiang Wang, Jiandong Yang, Yuping Tao, Shengfei Zhang, Xinmin Feng, Caifeng Yan

**Affiliations:** 1 Department of Orthopedics, Northern Jiangsu People’s Hospital, Yangzhou, China; 2 Department of Orthopedics, First Affiliated Hospital of PLA General Hospital, Beijing, China; 3 Department of Nephrology, Northern Jiangsu People’s Hospital, Yangzhou, China; 4 Department of Endocrinology, Northern Jiangsu People’s Hospital, Yangzhou, China

Retraction of: *Scientific Reports* 10.1038/s41598-017-18670-7, published online 17 January 2018

The Editors are retracting this article.

After publication, concerns were raised with respect to the data extraction. Post-publication peer review has confirmed that there are a number of inconsistencies with the original data, in regard to both sample size and ethnicity. In addition, several samples were included that did not meet the inclusion criteria, and the distribution of genotypes in the control group were not tested for Hardy Weinberg Equilibrium. The Editors therefore no longer have confidence in the conclusions presented.

Liang Zhang, Xin Yin, Jingcheng Wang, Yongxiang Wang, Jiandong Yang, Yuping Tao, Shengfei Zhang, Xinmin Feng & Caifeng Yan agree with the retraction and its wording. Daolinag Xu did not respond to the correspondence about the retraction.

